# A Framework on Polarization, Cognitive Inflexibility, and Rigid Cognitive Specialization

**DOI:** 10.3389/fpsyg.2022.776891

**Published:** 2022-03-24

**Authors:** James Shyan-Tau Wu, Christoph Hauert, Claire Kremen, Jiaying Zhao

**Affiliations:** ^1^Institute for Resources, Environment and Sustainability, University of British Columbia, Vancouver, BC, Canada; ^2^Department of Mathematics, University of British Columbia, Vancouver, BC, Canada; ^3^Department of Zoology, University of British Columbia, Vancouver, BC, Canada; ^4^Biodiversity Research Centre, University of British Columbia, Vancouver, BC, Canada; ^5^Department of Psychology, University of British Columbia, Vancouver, BC, Canada

**Keywords:** cognitive inflexibility, belief updating, conflict mindset, depolarization, social cohesion

## Abstract

Polarization is pervasive in the current sociopolitical discourse. Polarization tends to increase cognitive inflexibility where people become less capable of updating their beliefs upon new information or switching between different ways of thinking. Cognitive inflexibility can in turn increase polarization. We propose that this positive feedback loop between polarization and cognitive inflexibility is a form of threat response that has benefited humans throughout their evolutionary history. This feedback loop, which can be driven by conflict mindset, group conformity, and simplification of information, facilitates the formation of strong bonds within a group that are able to eliminate threats and increase individual fitness. Although cognitive inflexibility is conventionally seen as maladaptive, here we argue that cognitive inflexibility may be an adaptation under polarization. That is, in a highly polarized society most people only interact with members of their own social group, without having to confront perspectives from another group or interacting with out-group members. In this context, cognitive inflexibility creates rigid cognitive specialization, a set of cognitive traits that allow people to operate efficiently within their social circles but not outside of it. Although rigid cognitive specialization benefits individuals in the short term, it may lead to more polarization over the long run, and thus produce more conflict between groups. We call on future research to examine the link between cognitive inflexibility and rigid cognitive specialization.

## Introduction

Polarization is defined as the process in which two entities (individuals or groups of people) move toward opposite extremes of a continuum of viewpoints or opinions. Polarization has become alarmingly pervasive in today’s society, most notably in sociopolitical discourse (Jung et al., 2019). For example, liberals and conservatives are generally moving farther away from each other on a variety of issues (e.g., climate change, COVID-19, and immigration), and interactions between the two sides are marked by ideological conflict, mutual hostility, and lack of agreement (Ennser, 2012; Harel et al., 2020). Due to the prevalence of polarization, there has been great interest in understanding the cognitive properties of highly polarized people. One recent finding is that highly polarized people tend to be cognitively inflexible (Zmigrod et al., 2019), which is defined as an inability to alter thinking or update beliefs to accommodate new information and adapt to novel environments. In other words, highly polarized people tend to stick to existing beliefs and habits, sometimes to an extent that is actively detrimental to their wellbeing (Zmigrod, 2020). Because of this, cognitive inflexibility is usually considered maladaptive, as the ability to alternate between different styles of thinking (i.e., cognitive flexibility) is crucial for many tasks, such as empathizing (Yan et al., 2020) and cooperating with other people (Roy and Dugal, 1998) that require a person to switch between different viewpoints. Cognitive flexibility was also important in the evolutionary history of humans, as it facilitated the development of novel tools and survival strategies (Lombard, 2016).

However, here, we argue that under some circumstances, it may be more advantageous to be cognitively inflexible (i.e., sticking only to what aligns with a person’s views and often by extension their group affiliation), because in the context of a polarized society, there is often a heightened need to appease in-group members, and less of a desire to understand or interact with out-group members. Such actions tend to drive people to adopt specific inflexible cognitive traits depending on what social group they belong to. These inflexible traits, collectively, are defined as the person’s rigid cognitive specialization. As of now, there is little empirical evidence for rigid cognitive specialization, but some authors have speculated about its existence (Bertolotti and Magnani, 2017; Werner, 2021). Rigid cognitive specialization is an adaptation that allows an individual to thrive in a given group. Rigid cognitive specialization also allows the group to operate more efficiently and maintain social cohesion, which eventually results in better fitness for its members. Under polarization, some people may feel threatened by other groups with different viewpoints, and rigid cognitive specialization allows these people to block out conflicting opinions and find comfort in the similar views held by their in-group members. However, rigid cognitive specialization is not useful when groups with different views interact or cooperate with one another. It may generate more polarization between groups, exacerbate conflict between groups, and ultimately lead to a less stable society. In short, rigid cognitive specialization can be an adaptation for interactions between in-group members in the short term, but a maladaptation for interactions between out-group members in the long term.

## An Evolutionary Framework for Polarization and Cognitive Inflexibility

Humans are social animals whose chances of survival are greatly enhanced if they belong to a strong, unified group that is ready to confront perceived threats (Van Vugt and Schaller, 2008; Lo and Zhang, 2021). These threats could be natural (e.g., environmental disasters) or manmade (e.g., enemy tribes). Polarization, both in the short term and over an evolutionary timespan, is an important form of threat response (Boyer et al., 2015; McDermott and Anf Hatemi, 2018; Jutzi et al., 2020). Under polarization, members of a group can quickly rally to tackle a common threat. In past societies, this tendency increased the physical fitness of both the group and its individual members. In the current society, although physical survival is no longer a problem in most contexts, affiliation with a strong group in the face of a threat is still beneficial in that it can lead to a greater sense of psychological security and wellbeing, even if that threat is only perceived (Watson-Jones and Legare, 2016; Hart, 2019).

**Figure 1:**
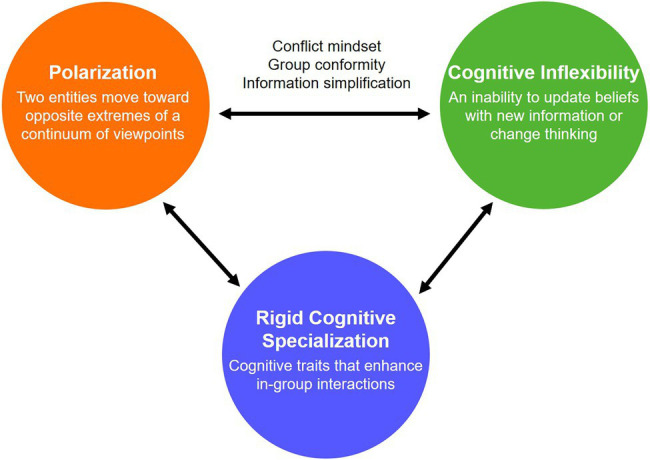
Bidirectional relationships between polarization, cognitive inflexibility, and rigid cognitive specialization.

Polarization is not a prerequisite for cognitive inflexibility, but it often creates favorable conditions for cognitive inflexibility to emerge. It is also worth noting that the relationship between polarization and cognitive inflexibility is likely bidirectional. That is, while people experiencing polarization tend to become cognitively inflexible, people who are more cognitively inflexible are more easily polarized (Zmigrod, 2020; van Baar and FeldmanHall, 2021). From an evolutionary perspective, this suggests that polarization and cognitive inflexibility may form a positive feedback loop, which ultimately favors the formation of strong, unified groups. With this in mind, below, we identify three mechanisms that are important for this feedback loop: group conformity, conflict mindset, and information simplification. Fundamentally, these mechanisms are independent. For example, group conformity can occur in the absence of the conflict mindset, and vice versa. However, these mechanisms can have complex interactions in the aforementioned feedback loop between polarization and cognitive inflexibility (see Figure 1).

### Group Conformity

Polarization, as a threat response, tends to heighten individuals’ desire to belong to a group, or “take a side” (Gross and De Dreu, 2019), and eventually leads the group to be more ideologically homogenous than their pre-polarized state (Sunstein, 1999). For example, a group of initially liberal leaning but ideologically diverse people tends to become more similar in their beliefs when faced with a conservative adversary (Sunstein, 1999). This shift to a homogenous state occurs because in order to effectively deal with a threat, the group needs to coordinate their actions efficiently, be united and cohesive, and cooperate to solve problems, often at the expense of individuality (Gelfand et al., 2020). Thus, the need for group affiliation under polarization often becomes group conformity where people fall in line with the majority opinion in a group (Siedlecki et al., 2016; Krueger et al., 2017). Group conformity in turn is linked to cognitive inflexibility, because individuals are bound by their group affiliations and unable to think for themselves (Zmigrod, 2020).

One mechanism behind group conformity is prestige bias, which is the adoption of behaviors or traits that exhibit prestige (e.g., popularity and endorsement by in-group members). The assumption of prestige bias is that the adoption of competent behavior is more likely to benefit a person’s wellbeing. However, because competence is often difficult to assess directly, people tend to rely on indirect cues which are collectively known as prestige (Jiménez and Mesoudi, 2019). These indirect cues were important for human survival in the past. For example, if a group leader is perceived to be popular and well respected, it is a sign that the leader is probably competent, and following them will increase one’s chances of survival. However, under polarization prestige bias often becomes an end as opposed to a means to an end, which means that people adopt certain beliefs simply because they want to belong to a certain group, sometimes going as far as to feign support for certain views just to be perceived favorably (Sunstein, 1999).

### Conflict Mindset

Polarization, by its definition, implies animosity between different groups of people (e.g., a protagonist and antagonist). As such, it is easy for people in a polarized group to adopt the conflict mindset (Stern and Kleiman, 2015). The conflict mindset is the tendency to see one’s own goals as unattainable without the exclusion of another person or group by which they feel threatened (Staw et al., 1981; De Dreu and Nijstad, 2008). The conflict mindset is one of the central components of threat response. Under the conflict mindset, the person or the group tends to divert their cognitive resources toward conflict-related tasks, which can be simply ignoring the threat and seeking closure, or devising new tools (e.g., weapons and technology) and strategies (e.g., rallying the masses with nationalism and creating a pro-conflict media narrative) to neutralize the perceived threat. For example, in the political divide between American liberals and conservatives, both sides are overly focused on discrediting the other side instead of seeking common ground (Heltzel and Laurin, 2020). People under the conflict mindset may show surprising creativity in conflict-related tasks (Miron-Spektor et al., 2011). However, they also tend to be more ossified in their thinking and less willing to consider non-conflict solutions, such as reconciling or cooperating with their perceived enemy (De Dreu and Nijstad, 2008). Moreover, group conformity and homogenization can be exacerbated by the presence of perceived enemies under a conflict mindset. For example, Ukraine President Zelensky’s approval ratings have soared to over 90% compared to 31% before the Russian invasion (The New Statesman, 2022). These conflict-induced traits also fit the definition of cognitive inflexibility, because they exhibit an inability to adopt alternative ways of thinking. This conflict-induced cognitive inflexibility can in turn increase polarization.

### Information Simplification

Polarization causes people to simplify the world in terms of discrete groups or categories with little middle ground. This can be useful when responding to a threat, where a group is required to make quick and decisive decisions without considering subtleties (Staw et al., 1981; Smith, 1988). Information simplification often occurs when inhibition overpowers updating (Buechner et al., 2021). Updating is the ability to modify existing beliefs based on new knowledge which is a hallmark of cognitive *flexibility*. On the other hand, inhibition is the ability to filter out information that contradicts with prior beliefs, while selectively attending to information that confirms prior beliefs (Miyake et al., 2000). One consequence of the directional filtering of information under inhibition is rationalization, which seeks coherence between decisions and beliefs in the service of justifying group actions (Heine et al., 2006; Qiu et al., 2020; Yong et al., 2021). From an evolutionary perspective, rationalization helped people cope with environmental stressors. Similarly, under polarization, an individual may deliberately misinterpret facts in a way that concurs with their existing beliefs, instead of modifying their beliefs to better represent reality (Golman et al., 2017; Sharot and Sunstein, 2020). For example, when presented with the same climate data, a climate alarmist and a climate denier tend to interpret the data differently according to their prior views on climate change (Luo and Zhao, 2019). When a group responds to a threat, information simplification makes it easier for group members to follow their leader’s directions and act as a collective against the threat. One current example of this phenomenon is in Russia where the government simplifies the information on news media in a way that drums up support for actions against Russia’s perceived threats, such as a pro-EU Ukraine or the West (Schimpfossl and Yablokov, 2014; Nygren et al., 2018); the same phenomenon can be seen in the coverage of Russian news outlets regarding the 2022 invasion of Ukraine. Information simplification leads to cognitive inflexibility which can in turn cause people to simplify the information into discrete categories (e.g., right or wrong) that can increase polarization.

### An Integrative View

We propose that the positive feedback loop between polarization and cognitive inflexibility is a form of threat response that has been beneficial to humans over the course of their evolutionary history and is still quite useful in some aspects today. By contrast, cognitive flexibility might not be useful in the current polarized society (Rand et al., 2017). While cognitive flexibility allowed human ancestors to devise novel ways of survival, survival in the physical sense is frequently less of an issue in the current society. Therefore, cognitive flexibility may have less pronounced consequences for fitness these days. Moreover, in a polarized society divided along many dimensions, such as politics and socioeconomic status, most people interact mostly with people in their own social group. This might further reduce the usefulness of cognitive flexibility, at least in terms of understanding how people outside of the social group think. For example, a liberal individual has little incentive to understand the way a conservative individual thinks, and vice versa, because there is little meaningful social interaction between these groups.

## From Cognitive Inflexibility to Rigid Cognitive Specialization

An important note about highly polarized people is that although they are cognitively inflexible, they may not be cognitively alike in other regards (Buechner et al., 2021). Inflexibility merely refers to the inability to alter existing beliefs based on new information, but it says nothing about what those beliefs are, much less what cognitive traits those beliefs correspond to. For example, many studies have found significant differences in how liberals and conservatives process information (i.e., cognitive style). Generally speaking, liberals tend to be more sympathetic to strangers, less sensitive about threats, and more emotionally troubled by inequality than conservatives (Napier and Jost, 2008; Acerbi et al., 2009; Waytz et al., 2019). In a polarized society, these differences could be reinforced by group norms, as well as the lack of meaningful interactions with out-group members. For example, a liberal individual simply needs to be capable of dealing with a predominantly liberal social group and does not need to understand or empathize with conservative views, and vice versa. From this perspective, cognitive inflexibility becomes more of an adaptation for an individual to survive in their social group. When an adaptation becomes sufficiently tailored for a certain environment, it becomes a specialization. Thus, we propose describing this type of cognitive inflexibility as rigid cognitive specialization.

Cognitive specialization refers to an individual being cognitively proficient at certain tasks, and it does not always correspond to cognitive inflexibility (DeCasien and Higham, 2019). The term specialization is borrowed from evolutionary biology. A classic example of specialization in animals is Darwin’s finches, which are a group of 18 species of birds that inhabit the Galapagos Islands. The most notable difference between the finches is their beak size and shape which is the result of becoming highly adapted for feeding on different food sources (Darwin, 1859). In the human contexts for example, scientists use the scientific method to address a question, and every field has certain scientific protocols to abide by. In this sense, scientists are cognitively specialized to a certain set of skills but are not inflexible, as they also need to learn new skills and explore new perspectives, both of which require cognitive flexibility.

By contrast, rigid cognitive specialization is when an individual is cognitively specialized but also cognitively inflexible. In principle, these people should be competent at certain tasks but are unable to adjust to a new task. Some journalists who work for highly politically biased media outlets provide an example of rigid cognitive specialization. They are specialized at writing stories that fit their organization’s worldview but are unable to break away from partisan thinking in their stories; hence, they are cognitively inflexible. In a sense, people who exhibit rigid cognitive specialization are similar to Darwin’s finches: the finches have become so specialized in exploiting a certain niche that they are unlikely to thrive outside it.

In the current sociopolitical climate, however, most people tend to interact with other people of similar views in their niche rather than people of opposing views. In this context of in-group interactions, rigid cognitive specialization can be an adaptation and an efficient way of navigating their social niches, which is limited in scope due to polarization. For example, rigid cognitive specialization for a liberal-leaning individual means falling in line with typical liberal arguments and the reasoning behind those arguments, or adopting and reinforcing liberal cognitive traits (e.g., openness to new experiences and empathy to strangers). By reinforcing these traits, rigid cognitive specialization may further increase the actual or perceived differences between social groups, and in turn further facilitate the polarization-cognitive inflexibility feedback loop discussed in the previous section (Figure 1).

On a longer timescale, we believe that cognitive specialization gradually increased throughout the evolutionary history of human beings. This is partly a response to societal specialization, which is a broad term encompassing all the dimensions along which civilization has divided into different categories, such as the division of labor (Henrich and Boyd, 2008; Cooper and West, 2018). In prehistoric societies, most people had to do a little of everything, such as hunting, gathering, building houses, and ceremonial duties, whereas in the current society, these tasks are usually allocated to their respective professions. The specialization of science is yet another example: scientists in the past such as Galileo, Goethe, and Newton were often polymaths, having a cursory understanding of a variety of fields, while the scopes of most scientists these days are much narrower, albeit much deeper and more focused.

In the current society, polarization has played a role in increasing societal specialization. For example, consider how polarization corresponds to the fragmentation of news media. In the past, most people consumed the same news from the same set of major media companies, whereas now media companies tailor their content for a specific audience (Tewksbury, 2005). Because of increasing polarization, and thus societal specialization, it is reasonable to assume that cognitive specialization also increased with polarization because of the distinct cognitive demands of the disparate groups. When cognitive specialization increases to a certain point, cognitive flexibility becomes less useful and costlier due to the decreased likelihood of having to switch between different ways of thinking. This is when the specialized groups become cognitively inflexible (i.e., rigid cognitive specialization).

We propose that the relationships between polarization, cognitive inflexibility, and rigid cognitive specialization are bidirectional (Figure 1). That is, (i) polarization reinforces cognitive inflexibility *via* conflict mindset, group conformity, and simplification of information; (ii) cognitive inflexibility creates rigid cognitive specialization by increasing contact with in-group members and reducing contact with out-group members; and (iii) rigid cognitive specialization leads to more polarization in a positive feedback loop. This process can also occur in the reverse direction: (i) rigid cognitive specialization implies an overreliance on certain cognitive traits, which can increase cognitive inflexibility; (ii) cognitive inflexibility exacerbates polarization *via* conflict mindset, group conformity, and simplification of information; and (iii) finally, polarization creates favorable conditions for rigid cognitive specialization to emerge.

## Future Directions

We call for future research to examine how polarization drives rigid cognitive specialization, and whether this is mediated by changes in cognitive inflexibility, increased interactions with in-group members, or decreased interactions with out-group members. Specifically, we advocate three directions for future research. First, future studies can examine the relationship between a person’s cognitive inflexibility and mental wellbeing. Since rigid cognitive specialization (which is a kind of cognitive inflexibility) is postulated as being beneficial, it should increase a person’s wellbeing. Conversely, better wellbeing could reinforce a person’s reliance on rigid cognitive specialization. There are some studies that show that cognitively inflexible people demonstrate higher emotional wellbeing, perhaps because they are shielded from the burden of always updating their beliefs upon new information (Wojcik et al., 2015). Second, future studies can also examine the tension between the costs and benefits of rigid cognitive specialization. To be more precise, rigid cognitive specialization seems to be beneficial for individuals and groups in the short term: it promotes group solidarity, increases psychological security of its members, and protects against perceived threats. Conversely, rigid cognitive specialization reduces the need for cognitive flexibility, which is more useful for processing out-group perspectives. Therefore, in the longer term, rigid cognitive specialization might lead to stagnation, discourage individuality, and even encourage cult-like behavior. Furthermore, rigid cognitive specialization is harmful for society as a whole because it discourages cooperation and meaningful discourse between different groups. Linking individual-level measures (e.g., personal wellbeing and group solidarity) to large-scale measures (e.g., level of political discourse) can help find a balance between individual, group, and societal wellbeing. Moreover, future studies can examine the scale of society as an important factor in determining the usefulness of rigid cognitive specialization. For example, in past smaller societies, most people interacted with a small number of in-group members, and rigid cognitive specialization offered a lot of advantages to people within a group, while its defects were mostly non-existent. However, in the current globalized society with fragmented groups with different views, people feel threatened by other groups, while at the same time, there is a need for different groups to cooperate. In this context, rigid cognitive specialization provides security for members within a given group, but at the expense of societal stability. It is simultaneously beneficial in some aspects (e.g., providing security to in-group members) while detrimental in others (e.g., preventing cooperation and causing conflict between groups). Third, future studies should design interventions aimed at depolarization. For example, systematically exposing people to opposing views can sometimes lead to depolarization and encourage meaningfully interaction with people of opposing views, but such interventions need to be meticulously designed, as they can easily backfire and increase polarization (Munson et al., 2013; Balietti et al., 2021).

Lastly, we must acknowledge that approaching social issues through an evolutionary lens has limitations. For example, evolutionary psychology is influenced by evolutionary biology which mostly addresses traits that are genetically transmissible (Smith, 2020). However, as of now it is not fully clear to what extent and *via* what mechanisms cognitive and behavioral traits in humans are genetically transmissible. Because of this, evolution in the biological sense may not be a perfect analogue for evolution of cognitive traits, behavior, or even culture. When addressing a large-scale evolutionary psychology question of polarization, there is a need to test the hypotheses mentioned above with multiple methods and data sources, such as lab experiments, cross-sectional surveys, demographic data, and archaeological records (Buss, 2019; Jung et al., 2019). Polarization poses enormous challenges to addressing global issues such as climate change, the pandemic, and wars. Effectively addressing polarization requires the mobilization and cooperation of all sectors of society. This research is urgently needed under the current sociopolitical polarization to provide insights on how to depolarize society, foster meaningful discourse, and preserve wellbeing of humanity.

## Data Availability Statement

The original contributions presented in the study are included in the article/supplementary material, further inquiries can be directed to the corresponding author.

## Author Contributions

JW wrote a draft of the manuscript with feedback from CH, CK, and JZ. JZ, CH, and CK edited the manuscript. All authors contributed to the article and approved the submitted version.

## Funding

CH acknowledges financial support from Natural Sciences and Engineering Research Council of Canada (NSERC) RGPIN-2021-02608.

## Conflict of Interest

The authors declare that the research was conducted in the absence of any commercial or financial relationships that could be construed as a potential conflict of interest.

## Publisher’s Note

All claims expressed in this article are solely those of the authors and do not necessarily represent those of their affiliated organizations, or those of the publisher, the editors and the reviewers. Any product that may be evaluated in this article, or claim that may be made by its manufacturer, is not guaranteed or endorsed by the publisher.
